# Population pharmacokinetics of peginterferon α2a in patients with chronic hepatitis B

**DOI:** 10.1038/s41598-017-08205-5

**Published:** 2017-08-11

**Authors:** Jingfeng Bi, Xingang Li, Jia Liu, Dawei Chen, Shuo Li, Jun Hou, Yuxia Zhou, Shanwei Zhu, Zhigang Zhao, Enqiang Qin, Zhenman Wei

**Affiliations:** 1 Research Center for Clinical & Translational Medicine, 302 Military Hospital, Beijing, 100039 China; 2 Department of Pharmacy, Beijing Tiantan Hospital, Capital Medical University, Beijing, 100050 China; 3 Laboratory Center, 302 Military Hospital, Beijing, 100039 China; 4 Infectious Disease Treatment Center, 302 Military Hospital, Beijing, 100039 China; 5 Ministry of Health, 302 Military Hospital, Beijing, 100039 China; 6 Medical Information Center, 302 Military Hospital, Beijing, 100039 China; 7 Department of Pharmacy, 302 Military Hospital, Beijing, 100039 China

## Abstract

There were significant differences in response and pharmacokinetic characteristics to the peginterferon α2a treatment among Chronic Hepatitis B (CHB) patients. The aim of this study is to identify factors which could significantly impact the peginterferon α2a pharmacokinetic characteristics in CHB patients. There were 208 blood samples collected from 178 patients who were considered as CHB and had been treated with peginterferon α2a followed by blood concentration measurement and other laboratory tests. The covariates such as demographic and clinical characteristics of the patients were retrieved from medical records. Nonlinear mixed-effects modeling method was used to develop the population pharmacokinetic model with NONMEM software. A population pharmacokinetic model for peginterferon α2a has been successfully developed which shows that distribution volume (V) was associated with body mass index (BMI), and drug clearance (CL) had a positive correlation with creatinine clearance (CCR). The final population pharmacokinetic model supports the use of BMI and CCR-adjusted dosing in hepatitis B virus patients.

## Introduction

Hepatitis B virus (HBV) associates approximately 780,000 deaths each year worldwide, mostly due to the chronic hepatitis B infection^1^. It has been proved that pegylated interferon alfa-2a (pegylated with a branched 40 kDa PEG chain) is an antiviral drug and it has a dual mode of action includes both antiviral and immunomodulatory effects^2^. The addition of polyethylene glycol to the interferon, through a process known as pegylation enhances the half-life of the interferon when we compared it to its native form^3^. This drug has been approved around the world, such as EU, U.S., China and many other countries, on the treatment of chronic hepatitis B (CHB).

Numerous international multi-centers randomized controlled clinical trials have proved that for the HBeAg-positive CHB patients, treated with peginterferon α2a 180 μg/week for 48 weeks and follow-up by 24 weeks observation, the HBeAg seroconversion rate was 32~36% and HBsAg seroconversion rate was 2.3~3%^4^. The significant difference in responses to the treatment among patients was observed^5, 6^. A preliminary study of the pharmacokinetics on peginterferon α2a in adults has indicated that the coefficient of variation (CV%) of AUC_0−t_ was 36.00%, t_1/2Z_ was 33.67%, T_max_ was 30.16%, and C_max_ was 36.60%^7^. We believed that the high inter-individual variability (IIV) of the pharmacokinetic characteristics may be the primary cause for the differences of curative effect.

Therefore, we aimed to identify the factors which could significantly influence the peginterferon α2a *in vivo* behavior in HBV patients. Furthermore, it is necessary to build a quantitative relationship between the influence factors and IIV. Population pharmacokinetic modeling was used to solve this problem^8^. Once this population model established, it will be helpful to realize precision medication for the patient with HBV.

## Results

### Patient demographics

The study of demography and clinical characteristics of the patients, which includes age (AGE, year), weight (WT, kg), body mass index (BMI, kg/m^2^), height (HT, cm), gender (GNDR, male = 1; female = 2) were retrieved from medical records. Laboratory results in records, such as serum creatinine (SCR, μmol/L), creatinine clearance (CCR, mL/min, estimated according to the Cockcroft-Gault formula^9^), aspartate transaminase (AST, U/L), alanine transaminase (ALT, U/L) and disease grade [Disease, hepatitis (APRI ≤ 2) = 1, Compensated Cirrhosis (APRI > 2) = 2] have been tested in one week before blood samples were collected. A total of 178 patients with 208 observations were obtained for analysis and the demographic background of patients for modeling was listed in Table 1.

**Table 1:** Demographic background and clinical characteristics of the subjects for modeling.

Characteristics	Number or mean ± SD	Median (range)
No. patients	178	—
No. observations	208	—
Observations per patient	1–4	—
Dose (ng)	156730.34 ± 27835.12	18000 (50000–180000)
Sampling time after dosing (h)	—	141.5 (15–13958)
GNDR, n (%)		
Male	99 (55.62)	—
Female	79 (44.38)	—
Age (year)	48.40 ± 12.91	50.5 (15–75)
Body weight (kg)	65.52 ± 11.74	64 (42.5–100)
Alanine transaminase (U/L)	34.79 ± 26.36	26.5 (3–150)
Aspartate transaminase (U/L)	37.65 ± 21.01	31 (14–149)
Creatinine clearance (mL/min)	91.39 ± 24.33	91.66 (44.80–166.87)
Serum creatinine (μmol/L)	74. 65 ± 12.42	73 (44–106)
Body mass index (kg/m^2^)	23.41 ± 3.48	23.33 (15.43–33.80)
Height (cm)	166.89 ± 7.95	168 (145–191)

**Figure 1:**
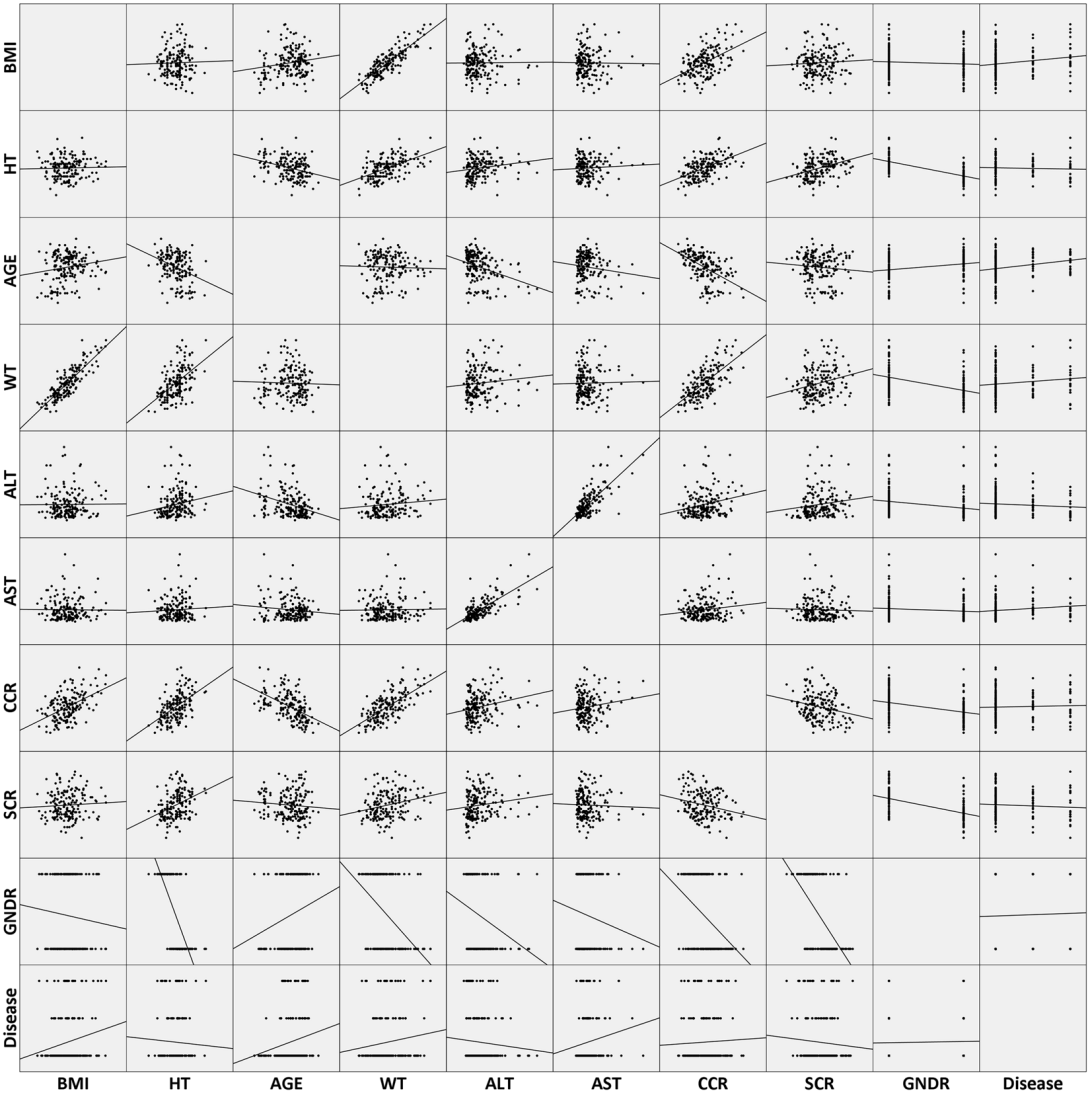
Relationship of all the candidate covariates. BMI: body mass index (kg/m^2^), HT: height (cm), AGE: age (year), WT: body weight (kg), ALT: alanine transaminase (U/L), AST: aspartate transaminase (U/L), CCR: creatinine clearance (mL/min), SCR: serum creatinine (μmol/L), GNDR: gender (male = 1; female = 2), Disease: disease grade [hepatitis (APRI ≤ 2) = 1, compensated cirrhosis (APRI > 2) = 2].

In order to identify the relationship of all the covariates, matrix diagram was performed by SPSS software (version 16.0, SPSS Inc., Chicago, IL, USA) and the result was shown in Fig. 1. Several significant were observed from the Fig. 1, such as AST and ALT, CCR and AGE, CCR and WT, GNDR and HT, BMI and WT, WT and HT, etc. These correlations indicated that related covariates may have interaction when adding them into the population model.

### Population pharmacokinetic model

**Figure 2:**
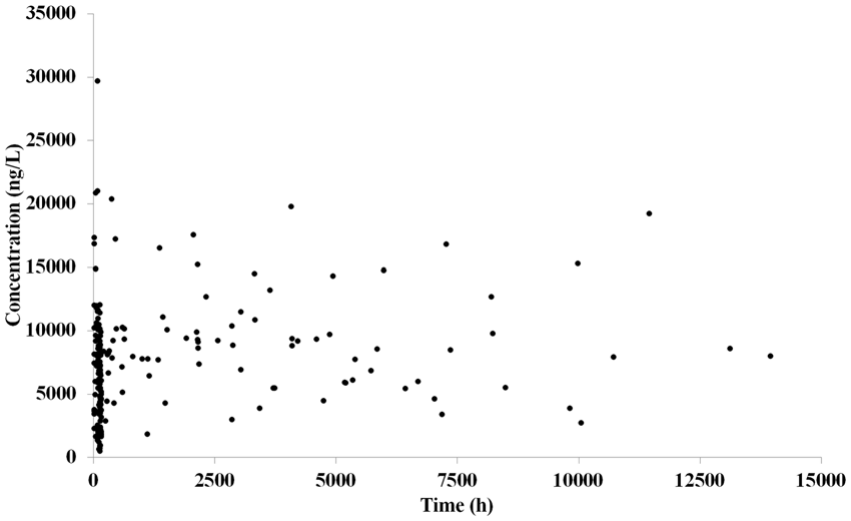
Scatter plot of drug concentration versus time. Each dot represents a data point.

The scatter plot of drug concentration versus time has been presented in Fig. 2. Due to the sparse data, it is difficult to identify the one- or two-compartmental model from this plot. Based on the objective function value (OFV) changes, the one-compartmental model was selected as the basic model. The following equations were used to describe this model:

1 $$\frac{{{\rm{dX}}}_{{\rm{a}}}}{{\rm{dt}}}=-{{\rm{K}}}_{{\rm{a}}}\times {{\rm{X}}}_{{\rm{a}}}\,[{{\rm{X}}}_{{\rm{a}}(0)}={\rm{Dose}}]$$

2 $$\frac{{\rm{dX}}}{{\rm{dt}}}={{\rm{K}}}_{{\rm{a}}}\times {{\rm{X}}}_{{\rm{a}}}-{\rm{CL}}\times {\rm{C}}\,[{{\rm{X}}}_{(0)}=0]$$

3 $${\rm{C}}\,=\,\frac{{\rm{X}}}{{\rm{V}}}$$

where X_a_ and X respectively represent the drug amount in absorption compartment and the central compartment. K_a_ represents the drug absorption rate constant from the dosing site. C represents the plasma drug concentration and V represents the distribution volume. X_a(0)_ and X_(0)_ respectively represent the initial drug amount in absorption and the central compartment.

After forward inclusion and backward elimination, BMI and CCR were included in the resulting population pharmacokinetic model, and the final model was described by the following equations (Equation 4–equation 6).

4 $${\rm{C}}{\rm{L}}=0.094\times {(\frac{{\rm{C}}{\rm{C}}{\rm{R}}}{91.39})}^{0.31}\times {{\rm{e}}}^{{\eta }^{1}}\,({\rm{L}}/{\rm{h}})$$

5 $${\rm{V}}=15.6\times {(\frac{{\rm{BMI}}}{23.41})}^{1.81}\times {{\rm{e}}}^{{\eta }^{2}}\,({\rm{L}})$$

6 $${{\rm{K}}}_{{\rm{a}}}\,=\,0.028\times {{\rm{e}}}^{{\eta }^{3}}\,(1/{\rm{h}})$$

In equation 4, the 0.094 (L/h) is the typical value of the CL, and 91.39 (mL/min) is the mean value of CCR. The 0.31 is the estimated coefficient which represents the relationship between the CCR and CL. In equation 5, 15.6 (L) means the V for an individual with the mean value of BMI (23.41 kg/m^2^), and 1.81 is the exponent between them (V and BMI). 0.028 (1/h) was the population typical value of K_a_ (equation 6), and no covariate had an effect on it to a statistically significant extent in this population. The parameter estimates of final population pharmacokinetic model were listed in Table 2. All parameters were estimated with acceptable precision [relative standard error (RSE) with the range from 14.75% to 28.58%, less than 30.00%]^10–12^.

**Table 2:** The parameters of final population pharmacokinetic model.

Parameter (unit)	Estimate	RSE%	95% CI^*^	IIV (CV%)	Bootstrap	
					Median	95% CI^#^
CL (L/h)	0.094	14.75	0.067–0.121	29.5	0.094	0.083–0.105
V (L)	15.60	16.78	10. 50–20.70	101.0	16.70	10.50–22.20
K_a_ (1/h)	0.028	28.58	0.012–0.044	64.0	0.033	0.014–0.065
CCR-CL	0.31	17.71	0.20–0.41	—	0.28	0.04–0.55
BMI-V	1.81	27.71	0.83–2.79	—	1.98	0.65–2.98
Residual error (proportional error, CV%, additive error, SD)						
CV%	19.4	—	—	—	20.2	14.6–31.5
SD (ng/L)	0.32	—	—	—	0.29	0.09–0.48

^*^The range was calculated by the equation estimate ± 1.96 SE.

^#^2.5th and 97.5th percentile of the ranked bootstrap parameter estimates.

### Model evaluation and validation

**Figure 3:**
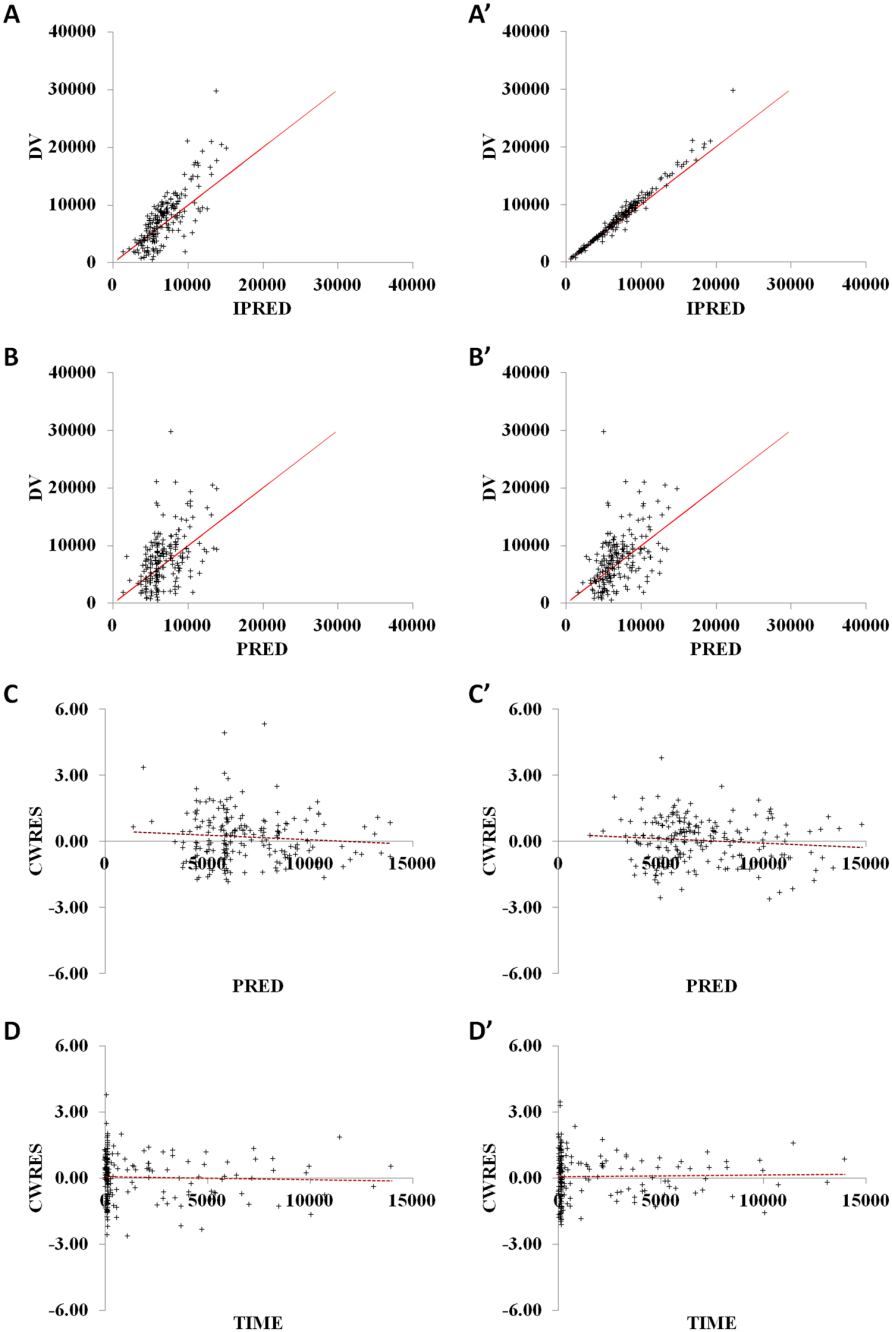
Goodness-of-fit of basic (**A,B,C** and **D**) and final (**A’,B’,C’** and **D’**) models. DV: dependent variable (observation); IPRED: individual prediction; PRED: prediction; CWRES: conditional weighted residuals. Solid lines represent identity lines and dashed lines mean zero lines. (**A** and **A’**): observation versus individual predictions; (**B** and **B’**): observation versus predictions; (**C** and **C’**): conditional weighted residuals versus predictions; (**D** and **D’**): conditional weighted residuals versus time.

**Figure 4:**
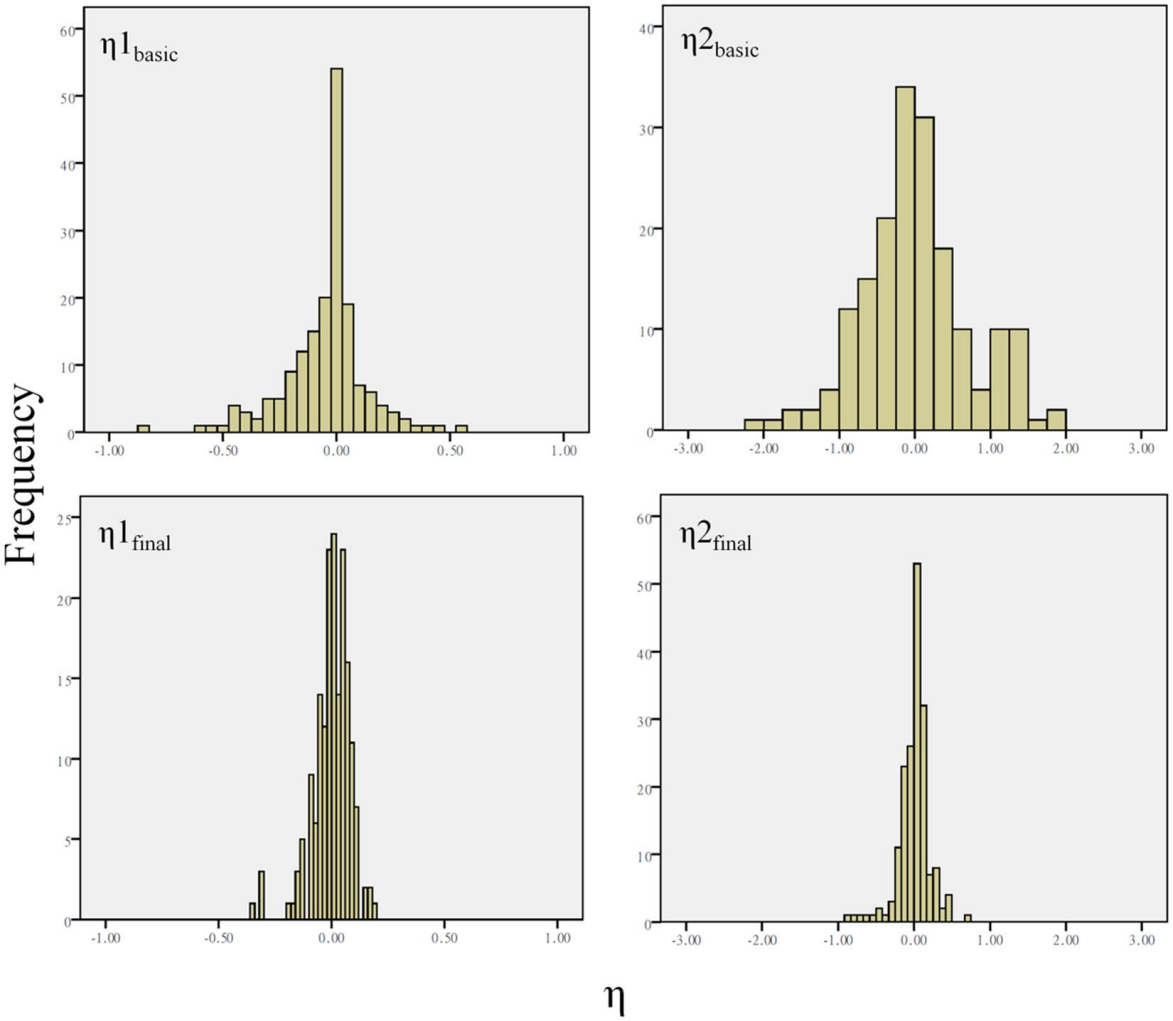
Distribution of η1 (IIV for CL) and η2 (IIV for V) for basic and final population models.

In order to assess the population pharmacokinetic model, the goodness-of-fit of basic and final models was displayed in Fig. 3 (basic model: 3A, 3B, 3C and 3D; final model: 3A’, 3B’, 3C’ and 3D’). Figure 3A and A’ verify the relationship between observation (dependent variable, DV) and individual prediction (IPRED). Compared with the Fig. 3A, a more precise relationship can be observed in Fig. 3A’. The scatter plots of DV versus prediction (PRED) were displayed in Fig. 3B and B’, and PRED of final model agrees well with DV. The diagnostic plots of conditional weighted residuals (CWRES) versus PRED and TIME (time after dose) show that CWRES in 3C′ and 3D′ are closer to zero line than 3C and 3D, indicating the final model fits the observations better. Two covariates were retained in the final model. Figure 4 displays the distribution of η1 (IIV for CL) and η2 (IIV for V) for basic (η1_basic_ and η2_basic_) and final (η1_final_ and η2_final_) population models. As the covariates were incorporated into the model, the variance of IIV for CL and V has become smaller. This indicates that part of IIV could be explained by the enrolled covariates.

**Figure 5:**
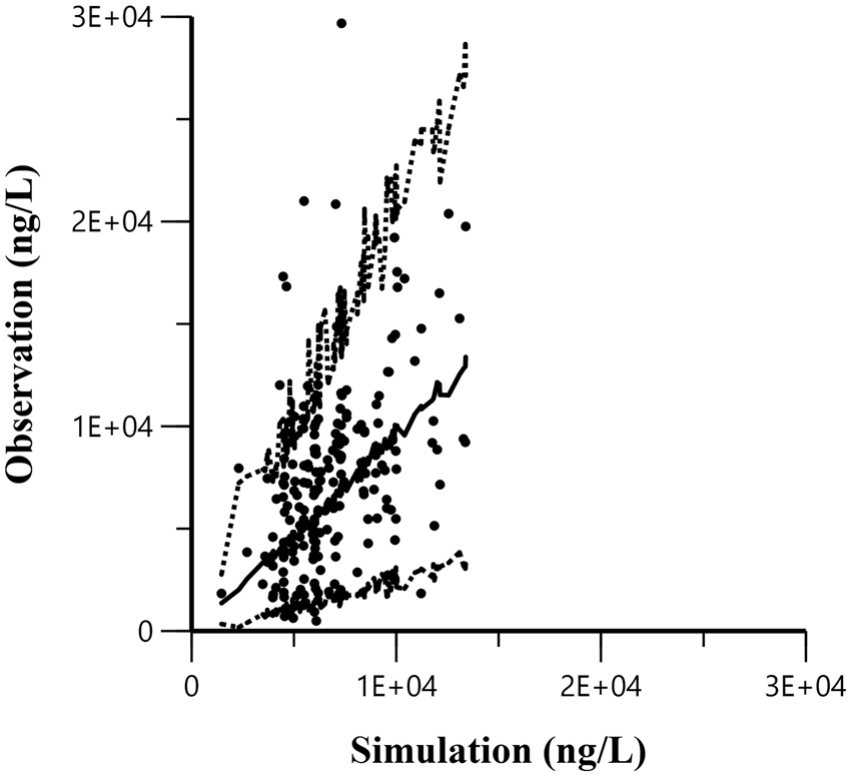
Visual predictive check plot of the final population model for drug concentration. Each dot means a data point; dotted lines are 5^th^ and 95^th^ percentiles and solid line are predicted 50^th^ percentile. The area between the 5^th^ and 95^th^ percentiles represents the 90% prediction intervals.

In addition, the bootstrap was also performed and the results were listed with the final estimates in Table 2. Our bootstrap analysis shows a successful rate of 94.6% (946 out of 1000 were successful in minimization). The 95% confidence interval (95% CI) of bootstrap analysis shows an acceptable robustness in the final population pharmacokinetic model. Peginterferon α2a visual predictive check (VPC) with the 90% prediction interval (90% PI) using the final population model overlaid with the actual original observations is shown in Fig. 5. The dashed lines are 5^th^ and 95^th^ percentiles and the solid lines are predicted 50^th^ percentile. The area between the 5^th^ and 95^th^ percentiles represents the 90% PI. About 90% of the original value lies within the 90% PI. The VPC plot infers adequate predictive properties of the final population model.

## Discussion

We aimed to investigate factors that may influence the pharmacokinetics of peginterferon α2a in the patient with HBV. However, the collected concentration data from the patients were random and sparse. The nonlinear mixed-effects modeling method is suitable for analyzing this kind of data set. A population pharmacokinetic model was developed in this study. The final model is a one-compartmental open model with first-order absorption, first-order elimination and exponential IIV on all the pharmacokinetic parameters. Our study verifies two major findings. First, the BMI-dependent increases in V. Second, CL decreases due to the decreases in CCR. The final population pharmacokinetic model supports the use of BMI and CCR-adjusted dosing in HBV patients. The data obtained from the sparse pharmacokinetic sampling may not contain enough information to estimate K_a_ accurately. Hence, the estimated RSE% of K_a_ (28.58%) was greater than CL (14.75%) and V (16.78%). Forward inclusion - backward elimination method was used to evaluate the effects of covariates, and no covariate was added into K_a_.

Schwarz *et al*. has reported the pharmacokinetics of peginterferon α2a in children with chronic hepatitis C. The final model is two-compartmental model^5^. Our data set was sparse and random, and it could not support multiple-compartmental model. According to the OFV reduction, one compartment model was the best choice for us. They also found the linear influence of body weight on the apparent volume of distribution in the central compartment^5^. BMI is defined as the body mass divided by the square of the body height, and it attempts to quantify the amount of tissue mass, such as muscle, fat, and bone. After BMI introduced into the V of the basic model, the reduction in OFV was 27.25. The incorporation of GNDR causes the decrease of 22.34 and WT induces the decrease of 4.66 in OFV. After BMI incorporated into the V, other covariates, such as WT, GNDR etc. did not influence V significantly (reduction of OFV less than 3.84). We believed that BMI has closer relative to V and it was selected as the final covariate to modify the parameter V.

Measuring SCR is a simple test and it is the indicator of renal function. However, SCR level can be influenced by many factors, such as gender, body weight, and age. Compared with SCR, CCR is a more precise indicator. Cockcroft-Gault formula^9^ is a commonly used surrogate marker to estimate the CCR. This formula, in turn, estimates glomerular filtration rate in mL/min. Based on the OFV change, CCR was retained in the CL of the final pharmacokinetic model. To the best of our knowledge, this is the first work shows that CCR had this significant influence on the CL. Part of this drug may be eliminated by the kidney. Nevertheless, this needs to be further investigated.

Jen *et al*. has reported the population pharmacokinetic analysis of peginterferon α2b. They concluded that body weight had a modest positive effect on the clearance^13^. Xu *et al*. also developed the population pharmacokinetics of peginterferon α2b in pediatric patients with chronic hepatitis C, and the final model indicating age-dependent increases in clearance and volume of distribution^14^. Peginterferon α2a (40-kDa peg conjugated to interferon α2a) and peginterferon α2b (12-kDa linear peg moiety conjugated to interferon α2b) have different molecular weight. Consequently, the pharmacokinetic properties between peginterferon α2a and peginterferon α2b are barely comparable.

There are several limitations in our study. (1) Only 208 concentration data among 178 patients were collected. For the most of the enrolled patients, only one drug concentration was obtained. The limited data may restrict the reliability of the final model. (2) The drug efficacy was the most important information in the clinic. However, only 47.8% (85/178) of patients’ drug efficacy was available. This made it difficult to develop the pharmacokinetic-pharmacodynamic (PK-PD) model. (3) IL28 plays a role in immune defense against viruses^15^, and it may have a significant impact on the drug efficacy. In our further study, information about drug efficacy will be collected to support the PK-PD analysis.

In summary, a population pharmacokinetic model for peginterferon α2a in patients receiving multiple subcutaneous doses has been successfully developed. Our model provides a useful tool that can be applied to estimate individual CL and V for the HBV patients, and to adjust dosing regimens with covariate factors (BMI and CCR).

## Methods

### Clinical trial registry

This study was registered on July 12^th^, 2013 in Chinese Clinical Trial Registry (A register participated in the WHO International Clinical Trial Registry Platform, ChiCTR-RO-13004320) and conducted from October 2013 to June 2016.

### Patient and treatment

Institutional Review Boards of 302 Military Hospital of China approved the study and written informed consents were obtained from all participants according to local regulations (for the patients under the age of 18, written informed consents were obtained from his/her guardian too). The study had been done in accordance with the principles of the Declaration of Helsinki and Good Clinical Practice. All authors had accessed to the study data, critically reviewed the manuscript at each draft, and approved the final draft for submission. The study protocol can be found in the Supplementary Information. 

Individuals who were considered CHB with interferon treatment indications of “Guide to chronic Hepatitis B Prevention, China, 2012”^16^, HBsAg positive, HBeAg positive, Anti-HBeAg negative, HBV DNA ≥ 10^5^ copy/ml, 2 × ULN ≤ ALT ≤ 10 × ULN, Serum total bilirubin ≤2 × ULN, age between 15 to 75, had not received any other antiviral therapy before this trial in past three months, could be enrolled into study. Any subjects that have one of these six features below are not qualified to this examination. (1) Any subjects have HCV or combine with either HDV or HIV; (2) Any subjects are taking other drug treatments which may have influences on the pharmacokinetics or pharmacodynamics of peginterferon α2a; (3) Any subjects have hepatic carcinoma and combine with either cardiac or renal or pulmonary or endocrine or blood or metabolic or gastrointestinal disease; (4) Any subjects are pregnant or lactating; (5) Any subjects had received other antiviral therapies in the past three months before this trial started; (6) Any subjects have poor compliance with medication. Peginterferon α2a was subcutaneously injected into patients once a week.

The T_max_ value of Peginterferon α2a is about 72 hours. In order to ensure all blood collection points evenly distributed at the absorption phase, near the peak concentration and distribution phase, every patient should be randomly assigned into three groups after administration with peginterferon α2a. Blood samples were collected within 48 hours, between 48 hours and 96 hours and after 96 hours. Specific blood collection time will be determined by research doctors after negotiating with patients. With patients’ consents, maximum four blood samples could be collected at different phases or different hospitalizations.

### Peginterferon α2a concentration assay

Peginterferon α2a concentrations in serum samples were analyzed using a commercial Human IFN-α Multi-Subtype ELISA Kit (product#41105) with a detection limit of 15 pg/mL manufactured by Pestka Biomedical Laboratories, Inc^17^.

### Basic pharmacokinetics model

Nonlinear mixed-effect modeling method was employed to develop the basic pharmacokinetic model for peginterferon α2a. All the plasma concentration-time data sets were fitted using the NONMEM software (Version 72, ICON Development Solutions, Ellicott City, MD, USA) with first-order conditional estimation with Interaction (FOCE-I) approach. IIV was described by an exponential variability model as follow^18^:

7 $${{\rm{P}}}_{{\rm{i}}}\,=\,{\rm{P}}\,\times \,{{\rm{e}}}^{{\rm{\eta }}i}$$

where P represents the typical value of parameter and P_i_ is the *i*th patient’s individual parameter. IIV is assumed to follow a log-normal distribution, and the random variable ηi is normally distributed with mean 0 and variance of ω^2^. Combined error model (proportional error and additive error) was used to calculate the residual error of the pharmacokinetic model:

8 $${{\rm{C}}}_{{\rm{ij}}}={{\rm{C}}}_{{\rm{ij}}}^{{\rm{P}}}\times (1\,+\,{{\rm{\varepsilon }}}_{1{\rm{ij}}})\,+\,{{\rm{\varepsilon }}}_{2{\rm{ij}}}$$

C_ij_^P^ and C_ij_ respectively represent model prediction and individual observation in *i*th patient’s *j*th concentration. ε_1_ characterizes the proportional error and is normally distributed with mean 0 and variances of σ_1_^2^. ε_2_ describes the additive error and is also distributed with mean 0 and variances of σ_2_^2^.

One- and two-compartmental open models with first-order absorption and elimination were applied to fit the data set. Model comparisons were made using the OFV for model discrimination, with the significance level of 0.05 (*df* = 2, ∆OFV = 5.99).

### Final pharmacokinetic model

Based on the basic pharmacokinetic model, candidate covariates, including AGE, WT, CCR, SCR, BMI, HT, GNDR, AST, ALT and disease grade on the basic model were investigated. For categorical covariates, such as GNDR and disease grade, they were incorporated using indicator variables. Other covariates were continuous and they were included into the model in the following ways:

9 $${{\rm{P}}}_{{\rm{i}}}\,=\,{\rm{P}}\,\times \,{(\frac{{\rm{COV}}}{{{\rm{MEAN}}}_{{\rm{COV}}}})}^{{\rm{\theta }}}\,\times \,{{\rm{e}}}^{{\rm{\eta }}i}$$

COV and MEAN_COV_ respectively represent the covariate and the mean value of this covariate. θ is the coefficient which represents the relationship between COV and P_i_. The effects of covariates were investigated using the forward inclusion-backward elimination approach. A forward inclusion was used and covariates with a decrease in the OFV by ≥3.38 (*P* = 0.05) were incorporated one at a time. After adding all the “significant” covariates from the forward inclusion in one model, the backward elimination step was performed. Covariates that caused a change ≥6.63 (*P* = 0.01) in the OFV when eliminated were kept in the model.

### Model evaluation and validation

The visual method was used to evaluate the basic and final population pharmacokinetic models. Scatter plots of DV versus IPRED, DV against PRED, the CWRES against PRED and TIME were drawn by Microsoft Office Excel 2007 software. Furthermore, the stability of the final model was assessed using the bootstrap technique. 1000 datasets were generated using the re-sampling method, and they were analyzed using the NONMEM software. After obtaining the mean and standard error of the fixed-effect parameters, the population estimates obtained from the final model were compared with the median and 95% CI of the bootstrap replicates. A predictive performance of the final pharmacokinetic model was examined by VPC. Simulations of 1000 virtual data sets were performed in the final population model. The median and 90% PI of simulated values were overlaid on the observed concentrations.

## Electronic supplementary material

Supplementary Information
