# Supplementary material for: Population pharmacokinetics of peginterferon α2a in patients with chronic hepatitis B
Source: Sci Rep. 2017 Aug 11;7:7893. doi: 10.1038/s41598-017-08205-5 (PMC5555209; doi:10.1038/s41598-017-08205-5)
Supplement: Supplementary file 1 — Supplementary Information [file 41598_2017_8205_MOESM1_ESM.pdf]

Title page

Title:

Population pharmacokinetics of peginterferon α2a in patients with chronic hepatitis B

Author list:

Jingfeng Bi[1#], Xingang Li[2#], Jia Liu[3#], Dawei Chen[4], Shuo Li[5], Jun Hou[1], Yuxia Zhou[6], Shanwei Zhu[7], Zhigang Zhao[2], Enqiang Qin[4] & Zhenman Wei[1]

1. Research Center for Clinical & Translational Medicine, 302 Military Hospital, Beijing, 100039, China.
2. Department of Pharmacy, Beijing Tiantan Hospital, Capital Medical University, 100050, China.
3. Laboratory Center, 302 Military Hospital, Beijing, 100039, China.
4. Infectious Disease Treatment Center, 302 Military Hospital, Beijing, 100039, China.
5. Ministry of Health, 302 Military Hospital, Beijing, 100039, China.
6. Medical Information Center, 302 Military Hospital, Beijing, 100039, China.
7. Department of Pharmacy, 302 Military Hospital, Beijing, 100039, China.

[#]These authors contributed equally to this work.

Corresponding authors:

Correspondence and requests for materials should be addressed to Z. W. (email: weizhenman@sina.com) or E. Q. (email: qeq2004@sina.com)

Protocol of 'Population pharmacokinetics of peginterferon α2a in patients with chronic hepatitis B'

1. Patients and treatment

All hospitalized cases will come from 302 Military Hospital of China, who are diagnosed with chronic Hepatitis B and treated with peginterferon α2a. Individuals were considered Chronic hepatitis B and in line with interferon treatment indications of "Guide of chronic Hepatitis B Prevention, China, 2012".

1.1 Inclusion criteria

1) HBeAg positive;
2) HBV DNA $\geq 10^5$ copy/ml;
3) 2×ULN≤ ALT ≤ 10×ULN
4) Serum total bilirubin ≤ 2×U LN
5) Aged from 15 to 75;
6) Not received other antiviral therapy 3 months before this trial;
7) Treated with peginterferon α2a not more than 24 months.

1.2 Exclusion criteria

1) Combined with HCV, HDV or HIV;
2) Receiving other drug treatment which may affect the pharmacokinetics or pharmacodynamics of interferon;
3) Combined with hepatic carcinoma, severe primary disease of heart, kidney, lung, endocrine, blood, metabolism and gastro intestine;
4) Pregnant or lactating women;
5) Received other antiviral therapy 3 months before this trial;
6) Medication compliance is poor.

1.3. Treatment

Peginterferon α2a was subcutaneously injected into patients once a week.

2. Methods

2.1 Blood collection point

The T_{max} of peginterferon α2a is about 72 hours. In order to ensure that the blood collection point evenly distributed at the absorption phase, near the peak concentration and distribution phase, every patient will be randomly assigned to

three groups after administration with peginterferon α2a: blood collection within 48 hours, between 48 hours and 96 hours and after 96 hours. Because the nonlinear mixed-effects model can fit the pharmacokinetic curve, specific blood collection time will be determined by research doctors according to the negotiation with patients. With the consent of the patient, multiple blood samples could be collected at different phases or different hospitalizations, but the maximum is 4 times.

2.2 Blood sample management

Collecting elbow vein blood 5 ml, centrifugal separation of serum, and stored in the -70 ℃ refrigerator to be tested. Peginterferon α2a concentrations in the serum samples were analyzed using a commercial Human IFN-α Multi-Subtype ELISA Kit (product # 41105) with a detection limit of 15 pg/mL manufactured by Pestka Biomedical Laboratories, Inc.

2.3 Clinical information collection

For the Clinical Laboratory has passed the ISO 15189 certification, clinical characteristics of the patients, including age, body weight, serum creatinine, creatinine clearance, body mass index, height, sex, aspartate transaminase, alanine transaminase and disease grade were retrieved from medical records. Laboratory results from the records are tested in one week before blood sample collection.

2.4 Statistical Analysis

2.4.1 Basic pharmacokinetics model

Nonlinear mixed-effect modeling method was employed to develop the basic pharmacokinetic model for peginterferon α2a. All the plasma concentration-time data set was fitted using the NONMEM software (Version 72, ICON Development Solutions, Ellicott City, MD, USA) with first-order conditional estimation with Interaction (FOCE-I) approach. IIV was described by an exponential variability model as follow:

$P_i = P \times e^{\eta i}$ Equation 1

where P means the typical value of parameter and P_i is the *i*th patient's individual parameter. IIV was assumed to follow a log-normal distribution, and the random variable ηi is normally distributed with mean 0 and variance of ω^2. Combined error model (proportional error and additive error) was used to calculate the residual error of the pharmacokinetic model:

$C_{ij} = C_{ij}^{P} \times (1+\varepsilon_{1ij}) + \varepsilon_{2ij}$ Equation 2

C_{ij}^{P} and C_{ij} represent model prediction and individual observation in *i*th patient's *j*th concentration, respectively. ε_1 characterizes the proportional error and is normally distributed with mean 0 and variances of σ_1^2. ε_2 describes the additive error and is

also distributed with mean 0 and variances of σ_2^2.

One- and two-compartmental open models with first-order absorption and elimination were attempted to fit the data set. Model comparisons were made using the objective function value (OFV) for model discrimination, with the significance level was selected a priori at 0.05 (df = 2, ΔOFV = 5.99).

2.4.2 Final pharmacokinetic model

Based on the basic pharmacokinetic model, effects of covariates, including age, body weight, serum creatinine, creatinine clearance, body mass index, height, sex, aspartate transaminase, alanine transaminase and disease grade on the basic model were investigated. For the categorical covariates, such as sex and disease grade, they were incorporated using indicator variables. The other covariates were continuous and they were included into the model in the following ways:

$$P_i = P \times \left(\frac{COV}{MEAN_{COV}}\right)^{\theta} \times e^{\eta i} \qquad \text{Equation 3}$$

COV and $MEAN_{COV}$ mean the covariate and the mean value of this covariate, respectively. θ is the coefficient representing the relationship between the COV and P_i. The effects of covariates were investigated using the forward inclusion-backward elimination approach. A forward inclusion was used and covariates with a change in the OFV of ≥ 3.38 (*P* = 0.05) were incorporated one at a time. After adding all the "significant" covariates from the forward inclusion in one model, the backward elimination step was performed. Covariates that caused a change ≥ 6.63 (*P* = 0.01) in the OFV when eliminated were kept in the model.

2.4.3 Model evaluation and validation

Visual method was used to evaluate the basic and final population pharmacokinetic models. Scatter plots of observation (dependent variable, DV) versus prediction (PRED), the conditional weighted residuals (CWRES) against PRED and TIME (time after dose) were drawn by Microsoft Office Excel 2007 software. Furthermore, the stability of the final model was assessed using the bootstrap technique. 1000 data sets were generated using the re-sampling method, and they were analyzed using NONMEM software. After obtaining the mean and standard error of the fixed-effect parameters, the population estimates obtained from the final model were compared with the median, 2.5% and 97.5% percentiles (95% confidence interval, 95%CI) of the bootstrap replicates.
